# Acute coronary syndrome with large thrombus successfully managed with no-stenting revascularization based on intravascular imaging in a patient with hyperhomocysteinemia: a case report

**DOI:** 10.1186/s13256-020-02531-5

**Published:** 2020-11-09

**Authors:** Keisuke Shoji, Kenji Yanishi, Noriyuki Wakana, Naohiko Nakanishi, Kan Zen, Takeshi Nakamura, Takeshi Shirayama, Satoaki Matoba

**Affiliations:** Department of Cardiovascular Medicine, Kyoto Prefectural University of Medicine, 465 Kajii-cho Kawaramachi-Hirokoji, Kamigyo-ku, Kyoto, 602-8566 Japan

**Keywords:** hyperhomocysteinemia, acute coronary syndrome, non-stenting revascularization

## Abstract

**Background:**

Hyperhomocysteinemia is caused by genetic and environmental factors, which can result in systemic arteriosclerosis and arteriovenous thrombosis including acute coronary syndrome. Thrombus burden in patients with acute coronary syndrome and hyperhomocysteinemia might involve the culprit lesion as compared with those without any coagulopathy. The primary percutaneous coronary intervention with stent implantation had been established as the treatment strategy for patients with acute coronary syndrome. However, in patients with acute coronary syndrome with high thrombus burden or uncontrolled coagulopathy, stent implantation might lead to slow-flow phenomenon or stent thrombosis. Therefore, the treatment strategy in these patients was not established.

**Case presentation:**

A 49-year-old Japanese man with history of splenic infarction of unknown cause had continued anticoagulant therapy since its diagnosis, but stopped taking the medication several months ago. He presented with sudden-onset chest dorsalgia. Contrast computed tomography showed a small pulmonary embolism and his troponin I level was elevated on initial laboratory test. Coronary angiography revealed a contrast defect caused by a large thrombus from the proximal to mid portion of the left anterior descending artery. Near-infrared spectroscopy–intravascular ultrasonography showed a large amount of thrombus without lipid plaque. Therefore, revascularization was performed using a thrombus-aspiration catheter and intracoronary thrombolysis. In addition, , hyperhomocysteinemia and a deep vein thrombosis occurred. He was diagnosed with acute coronary syndrome complicated with pulmonary embolism and deep vein thrombosis simultaneously induced by hyperhomocysteinemia. After 1 week of antithrombotic therapy, near-infrared spectroscopy–intravascular ultrasonography and optical coherence tomography revealed a decreased thrombus and no significant residual organic stenosis in the left anterior descending artery. He continued conservative therapy with antithrombotic medications including aspirin and warfarin and had no cardiovascular events after discharge. Follow-up coronary angiography and optical coherence tomography at 9 months revealed complete disappearance of the thrombus and no severe stenosis.

**Conclusions:**

Hyperhomocysteinemia should be considered as a cause of arterial vein thrombosis of unknown cause. The antithrombotic therapy and percutaneous revascularization without stenting based on intravascular imaging might be a safe and effective treatment option in patients with acute coronary syndrome complicated with hyperhomocysteinemia.

## Background

Homocysteine is an amino acid produced as an intermediate metabolite of methionine metabolism. Hyperhomocysteinemia is caused by genetic (enzyme deficits) and environmental (nutritional deficiencies, drugs, or toxins) factors, which can cause systemic arteriosclerosis and arteriovenous thrombosis [1–5]. Moreover, hyperhomocysteinemia was known to be associated with coronary artery disease (CAD) including acute coronary syndrome (ACS). In patients with ACS with coagulopathy such as hyperhomocysteinemia, the thrombus burden might possibly involve the culprit lesion more than in patients with ACS without any coagulopathy. The primary percutaneous coronary intervention (PCI) with stent implantation had been established as the treatment strategy for patients with ACS. However, in patients with ACS with high thrombus burden or uncontrolled coagulopathy, stent implantation might lead slow-flow phenomenon or stent thrombosis, and the treatment strategy for the cases was not established.

We encountered the case of patient with ACS with a huge thrombus simultaneously complicated with pulmonary embolism (PE) and deep venous thrombosis (DVT) induced by hyperhomocysteinemia. Here, we describe the good clinical outcome of antithrombotic therapy and percutaneous revascularization without stent based on intravascular imaging for ACS induced by hyperhomocysteinemia.

## Case Presentation

**Fig. 1:**
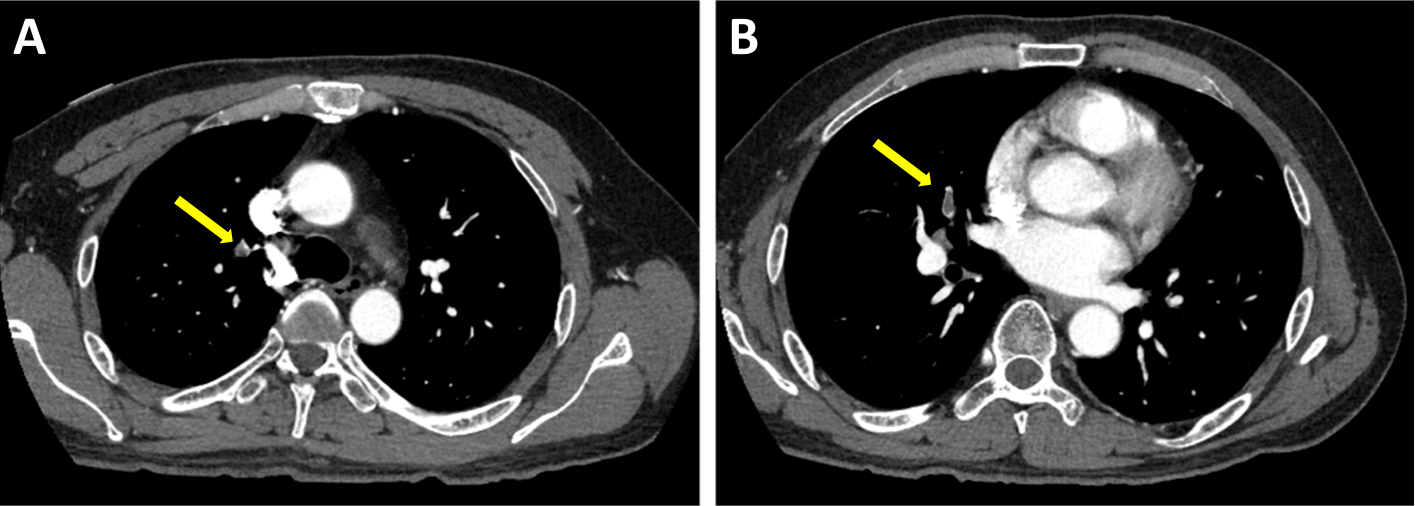
Contrast computed tomography showing small pulmonary embolism. Contrast computed tomography revealed small pulmonary embolism in the right segmental pulmonary artery (yellow arrow in **a** and **b**)

**Fig. 2:**
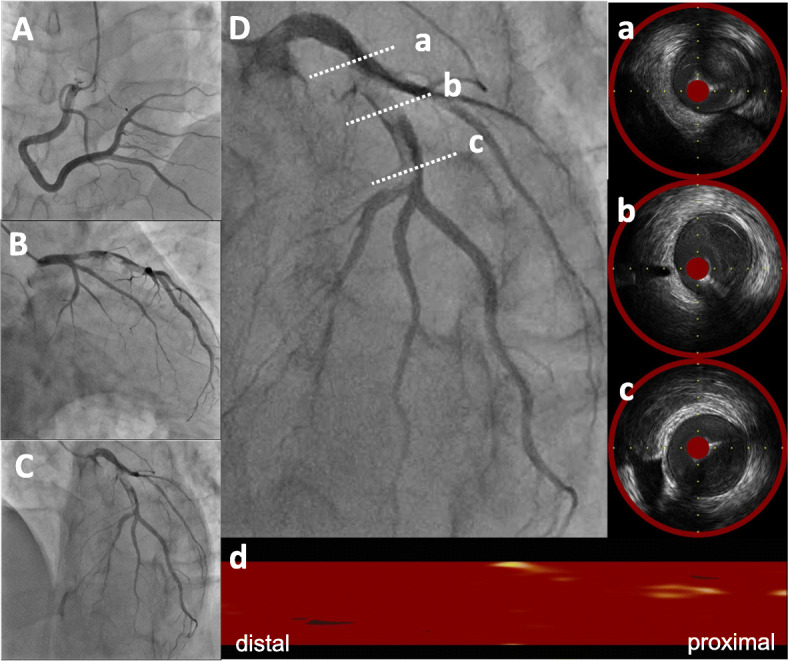
Baseline coronary angiography and near-infrared spectroscopy–intravascular ultrasonography. Coronary angiography (CAG) revealed severe stenosis from mid to proximal left anterior descending (LAD) artery and no severe stenosis in the right coronary artery and circumflex artery (**A-C**). Near-infrared spectroscopy–intravascular ultrasonography (NIRS–IVUS) findings in the culprit lesion showed a low echoic component suspecting thrombus continuing from mid to proximal LAD (**D**-a, b, c) and organic fibrous plaque behind the thrombus. The lipid plaque is shown as a yellow region on the chemogram. NIRS chemogram map presented the maximal lipid core burden index (4 mm) = 81 (**D**-d)

**Fig. 3:**
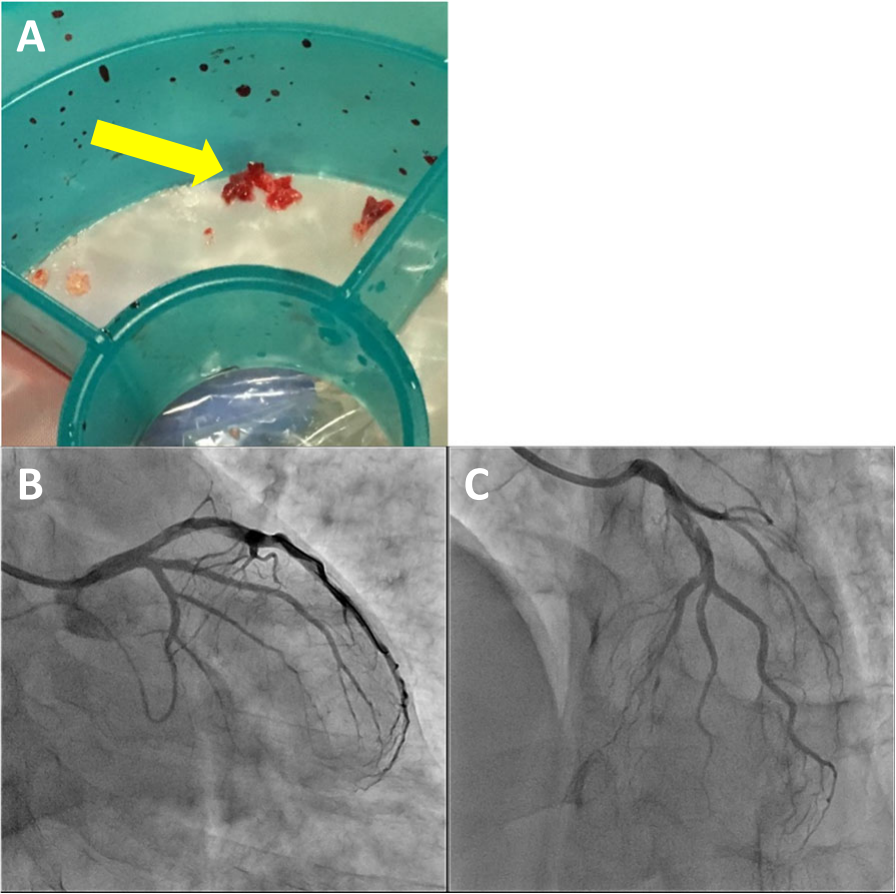
The collected thrombus using an aspiration catheter and final coronary angiography. The collected thrombus using a repeated aspiration thrombectomy (**a**, yellow arrow; thrombus). Final coronary angiography revealed thrombolysis in myocardial infarction (TIMI) grade 3 flow and reducing thrombus in the left anterior descending artery (**b, c**)

**Fig. 4:**
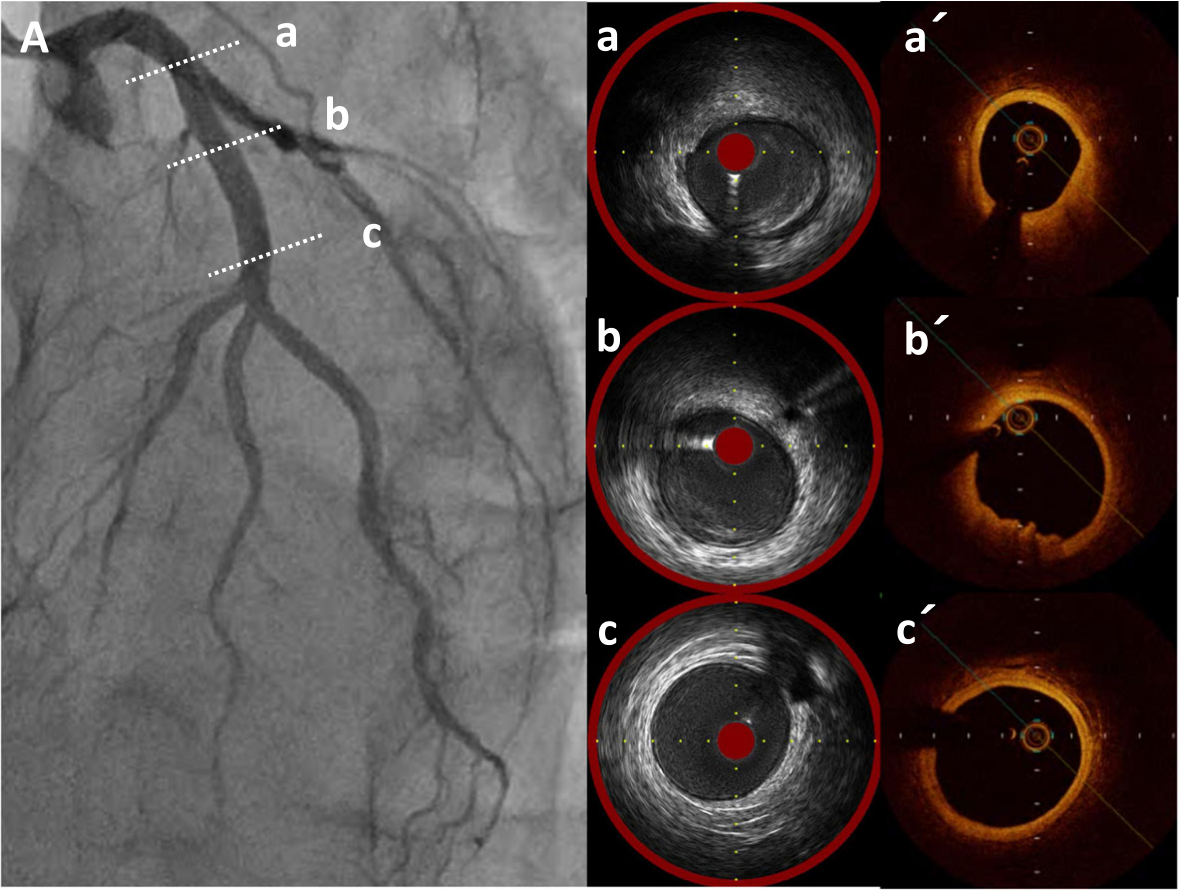
The findings of near-infrared spectroscopy–intravascular ultrasonography and optical coherence tomography in 1 week. Coronary angiography showed further reduced thrombus without severe stenosis in the left anterior descending (LAD) artery. The findings of near-infrared spectroscopy–intravascular ultrasonography (**A**-a, b, c) and optical coherence tomography (**A**-a´, b´, c´) revealed a small amount of residual thrombus in the short segment and mild fibrous plaque in the proximal left anterior descending artery

**Fig. 5:**
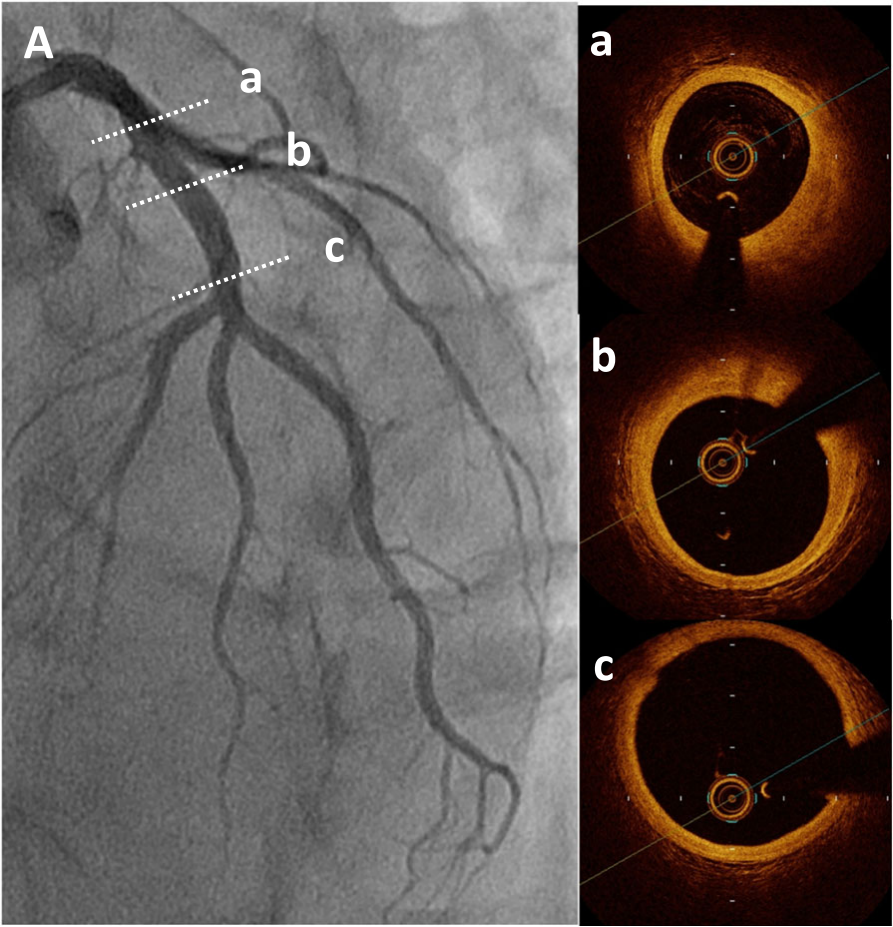
Coronary angiography and optical coherence tomography in 9 months. Coronary angiography showed no severe stenosis and no thrombus (**A**). Findings of optical coherence tomography (**A**-a, b, c) revealed mild fibrous plaque and complete withdrawal of thrombus in the proximal left anterior descending artery

A 49-year-old Japanese man had splenic infarction with unknown cause 2 years ago. He had continued to take warfarin as anticoagulant therapy, but stopped taking the medication as self-judgment several months ago. He had no classical arteriosclerosis factors (hypertension, dyslipidemia, diabetes mellitus, and smoking status). He had sudden-onset rest chest dorsalgia and was transported to our hospital. Immediately after arrival, his pain temporarily improved. On initial examination, his blood pressure was 130/83 mmHg, pulse rate 110 beats/min, respiratory rate 30 cycles/min, and oxygen saturation 100% on room air. His physical examination showed a regular heart rhythm and no murmur, normal respiratory sounds in both lungs, and no edema in his lower extremities. On initial laboratory test, his high-sensitivity troponin I level was 1453.1 pg/mL (normal range: 0.0–26.2), creatinine kinase (CK) 261 U/L (59–248), CK-MB 26 U/L (0–6), platelets 31.5 × 10^4^ /μL (15.8–34.8 × 10^4^), fibrinogen 237 mg/dL (190–390), and D-dimer 1.7 μg/ml (0–0.9). Initial electrocardiography showed normal sinus rhythm and no ST-T change. Transthoracic echocardiography revealed normal wall motion and no asynergy. Contrast computed tomography (CT) showed a small PE and no aortic dissection (Fig. 1a, b). However, he complained of chest pain again following CT. Emergent coronary angiography (CAG) showed no severe stenosis in the right coronary artery and left circumflex artery, but a large thrombus with Thrombolysis In Myocardial Infarction (TIMI) grade 3 flow from the proximal to mid portion of the left anterior descending (LAD) artery (Fig. 2a-c). A 7-Fr guiding catheter was advanced through the left radial artery to the left coronary artery ostium, and a guidewire was advanced to the distal LAD artery. Near-infrared spectroscopy–intravascular ultrasonography (NIRS–IVUS) showed a large amount of thrombus from the proximal to the mid LAD artery, and the max lipid core burden index (LCBI) was 81 (Fig. 2d). Thrombectomy was performed using a 7-Fr aspiration catheter (Fig. 3a) and intracoronary thrombolysis by urokinase 60,000 U. Angiography and NIRS–IVUS revealed decreased thrombus in the LAD artery (Fig. 3b-c). The procedure was completed at this time because additional angioplasty with or without stent was at high risk of slow-flow phenomenon. In the pathological findings, the collected thrombus using an aspiration catheter had many fibrin precipitates. We suspected paradoxical embolism because the ultrasonography revealed a DVT in the bilateral lower leg, but contrast CT and transesophageal echocardiography showed no right-to-left short circuit including a patent foramen ovale, atrial septal defect, and ventricular septal defect, and pulmonary arteriovenous fistula. Further examination of coagulation abnormality revealed hyperhomocysteinemia (total homocysteine level: 140.8 nmol/mL [6.3–18.9], vitamin B12: 121 pg/mL [180–914], folate: 2.3 ng/mL [> 4.0], protein C activity: 111% [70–140], and free protein S: 131.7% [60–150]). Therefore, he was diagnosed with ACS complicated with PE and DVT simultaneously induced by hyperhomocysteinemia. He continued antithrombotic therapy using aspirin, warfarin (his international normalized ratio was controlled from 2 to 3), and heparin (1.5–2.5 times the reference value activated partial thromboplastin time) for 1 week. Peak CK was 347 U/L, and NIRS–IVUS and optical coherence tomography (OCT) revealed decreased thrombus and no significant residual organic stenosis in the LAD artery following 1 week of antithrombotic therapy (Fig. 4a). He was discharged from our hospital continuing antithrombotic therapy with aspirin and warfarin. Follow-up CAG and OCT after 9 months revealed complete thrombus disappearance in the LAD artery (Fig. 5a). His total homocysteine level had decreased to 85.1 nmol/mL by the dietary therapy. He had no cardiovascular event or major bleeding for 1 year.

## Discussion and conclusions

Hyperhomocysteinemia was reportedly associated with systemic atherosclerosis [1, 2] and thrombosis [3] including CAD and stroke [4, 5]. Several studies suggested the possible mechanism of atherothrombosis including endothelial injury, platelet activation, smooth muscle proliferation, oxidative modification of low-density lipoproteins, endothelial-leukocyte interactions, inhibition of thrombomodulin activity, reduction of protein C activation, and elevated Factor VIIa, and thrombin generation [6, 7]. Hyperhomocysteinemia should also be considered in young patients with repeated thrombosis or cardiovascular events. Severe levels of hyperhomocysteinemia (> 100 μmol/L) might be caused by genetic enzyme deficiency of homocysteine metabolism [8]. Our patient was classified as severe hyperhomocysteinemia, possibly due to genetic enzyme deficits. However, additional examination including genetic tests could not be performed because patient consent was not obtained. Furthermore, he had an imbalanced eating habit and was expected to lack the vitamin intake required for homocysteine metabolism (folate, vitamin B12, and vitamin B6). The efficacy of homocysteine-lowering treatment on supplement with folate, vitamin B12, and vitamin 6 was controversial. Homocysteine-lowering treatment did not prevent cardiovascular events and recurrence of venous thrombosis, but could be harmful instead [9–11]. Therefore, our patient was not treated with supplements, but a meal guide was provided. Further studies are needed to determine whether normalization of homocysteine levels after cardiovascular events or thrombotic events is useful.

ACS is a life-threatening serious disease that needs immediate revascularization without major complications for good clinical outcomes. Intravascular imaging technology supports percutaneous revascularization and can provide information on the pathophysiology and complication risk. NIRS is an imaging diagnostic technique based on near-infrared spectroscopy. NIRS–IVUS can detect the lipid components through a near-infrared absorption pattern different from other tissues. Moreover, NIRS–IVUS can also evaluate vascular properties behind the thrombus or stent strut compared with OCT. The size of the lipid plaque is quantitatively identified as LCBI on the NIRS chemogram. A previous study reported that a max LCBI (4 mm) of > 400, as detected by NIRS–IVUS, was a signature of plaques causing ST-elevated myocardial infarction (STEMI) [12]. OCT is an imaging diagnostic technique that used light waves. OCT has a high resolution of 10 μm, which is about ten times that of IVUS. Compared with IVUS, OCT can distinguish various tissues of the vessel wall in detail according to luminance and degree of signal attenuation. However, the tissue depth in near infrared OCT is as small as approximately 2 mm, and in the presence of lipid plaque or thrombus, the tissues behind them cannot be visualized due to signal attenuation. Therefore, features of each intravascular imaging should be well understood. In our case, NIRS–IVUS findings in the primary intervention showed that the max LCBI (4 mm) from the proximal to mid LAD artery was very low, and mild organic stenoses were found behind the large thrombus. Therefore, we selected revascularization without stenting to prevent complications associated with stent placement. Moreover, 7 days later, NIRS–IVUS and OCT showed a small amount of residual thrombus and fibrous plaque with mild stenosis in the proximal LAD artery. Therefore, we did not perform additional revascularization according to intravascular imaging. Based on the NIRS–IVUS and OCT imaging results, the etiology of this case might be plaque erosion rather than plaque rupture.

PCI with stent implantation has been an established method of revascularization for ACS. However, thrombus formation is involved in ACS patients, which may cause major complications in the primary PCI with stent implantation. Distal thrombus embolization during PCI leads to coronary no reflow and increases the infarct size [13, 14]. Moreover, thrombus protrusion through stent struts has been associated with early stent thrombosis [15]. Considering these complications, the necessity of stent placement in patients complicated with coagulopathy should be carefully determined. Some patients with ACS complicated with hyperhomocysteinemia succeeded in angioplasty with or without stenting [16–19], but some failed due to the large clot [20, 21]. The EROSION study reported that conservative treatment with antithrombotic therapy without stenting for patients with ACS caused by plaque erosion might be a feasible and useful option [22, 23]. The no-stenting revascularization and conservative antithrombotic therapy (aspirin and warfarin) guided by intravascular imaging provided a 1-year good clinical outcome on our patients with ACS complicated with hyperhomocysteinemia.

Recent studies have failed to show the clinical benefits of PCI with routine aspiration thrombectomy in patients with ACS compared with PCI alone [24, 25]. However, these studies had several limitations such as inclusion of some patients with a small thrombus burden. Reducing thrombus burden through aspiration thrombectomy might be useful if stent-less revascularization is considered in patients with ACS with high thrombus burden. Moreover, the selective use of aspiration thrombectomy in patients with ACS with a large amount of thrombus burden induced by coagulation disorders might be more feasible and useful.

Generally, thrombolysis using urokinase in patients with ACS other than STEMI is not recommended [26, 27]. This is because the thrombus in patients with ACS other than STEMI is composed mainly of platelets and urokinase that promote platelet activities. However, the pathological findings of the collected thrombus in our case showed that it was a fibrinogen-based thrombus. Therefore, intracoronary urokinase might have contributed to the alleviation of thrombus burden. Conversely, in patients with ACS caused by plaque erosion who were managed conservatively without stenting, tirofiban (glycoprotein [GP] IIb/IIIa inhibitor) provided additional benefits in reducing residual thrombus without increasing the risk for bleeding [28]. GP IIb/IIIa inhibitors might also be an effective and feasible option even for ACS complicated with hyperhomocysteinemia. However, GP IIb/IIIa inhibitors are not available in Japan because their effectiveness was not yet proven in clinical trials [29]. Therefore, the effectiveness of thrombolytic therapy for ACS complicated with coagulopathy should be evaluated in future studies.

In conclusion, hyperhomocysteinemia should be considered in cases of repeated thrombosis and abnormal thrombotic tendency. Moreover, careful revascularization and antithrombotic therapy without stent under intravascular imaging guidance may be considered to have a good outcome in patients with ACS with hyperhomocysteinemia.

## Data Availability

All data generated or analyzed during this study are included in this published article and in its additional files.

## Abbreviations

ACS: Acute coronary syndrome
CAG: Coronary angiography
CAD: Coronary artery disease
CK: Creatinine kinase
CT: Computed tomography
DVT: Deep vein thrombosis
GP: Glycoprotein
LAD: Left anterior descending
LCBI: Lipid core burden index
NIRS-IVUS: Near-infrared spectroscopy–intravascular ultrasonography.
OCT: Optical coherence tomography
PCI: Percutaneous coronary intervention
PE: Pulmonary embolism
STEMI: ST elevated myocardial infarction
